# Nostril dominance at rest associated with performance of a left hemisphere-specific cancellation task

**DOI:** 10.4103/0973-6131.43542

**Published:** 2008

**Authors:** Sasmita Samantaray, Shirley Telles

**Affiliations:** Department of Yoga and Bioscience, Swami Vivekananda Yoga Anusandhana Samsthana, Bangalore, India

**Keywords:** Left cerebral hemisphere, letter cancellation task, nostril dominance

## Abstract

**Background:**

An association has been reported between the dominant nostril through which we breathe and the cerebral hemisphere found to be active.

**Aims:**

To understand the association between the nostril dominant at rest and the performance in a cancellation task using verbal information–a left hemisphere task.

**Materials and Methods:**

Two hundred eighty-nine normal, healthy volunteers attending a one week nonresidential yoga camp were assessed in a single 30 minute period. Nostril dominance was assessed using a standard method. After this, participants were given the letter cancellation task and nostril dominance was again checked. For each participant, the numbers of letters that had been left out and wrongly cancelled as well as total errors were assessed. The Mann-Whitney u test and Chi-Square test were used to assess whether there was a significant difference in cancellation task performance between right and left nostril-dominant persons.

**Results:**

There was no statistically significant difference between right and left nostril-dominant participants.

**Conclusions:**

The present results do not support previous findings of contralateral cerebral hemisphere improvement with breathing through a specific nostril.

## INTRODUCTION

The nasal cycle is an ultradian rhythm characterized by alternating patency of the left and right nostrils, with a periodicity of two to eight hours.[1,2] Some reports suggested a connection between the phases of the nasal cycle and the dominant cerebral hemisphere that is mediated through a neural reflex.[3]

This connection was based on studies using the electroencephalogram (EEG). Forced unilateral, nostril-breathing, modified EEG activity was monitored over the two hemispheres; greater EEG amplitudes were observed on the side contralateral to the patent nostril. The connection between the dominant nostril and cerebral functions was also believed to influence performance in specific tasks. In a study on 23 right-handed males, relatively greater cognitive ability in one hemisphere was found to correspond to unilateral, forced nostril-breathing in the contralateral nostril.[7] Cognitive performance ratios can be influenced by forcibly altering the breathing pattern.[4–7] Adult volunteers who exhibited right-nostril dominance during normal breathing, tended to perform better on simple perceptual tasks with verbal information, known to be carried out by the left hemisphere, compared with subjects whose left nostril was dominant.[6] Similarly, during the left nostril-dominant phase, subjects performed better on simple perceptual tasks using spatial information inferred to be carried out by the right hemisphere. Both verbal and spatial tasks involved deciding whether stimulus pairs were the same or different. For the verbal tasks, the stimuli were pairs of upper and lower case letters, while for the spatial task, the stimuli were pairs of random, seven-dot patterns. There was no significant effect of forced uninostril breathing on performance in these tasks. In contrast, a subsequent study of undergraduate students whose average age was 20.7 years, showed that forced left-nostril breathing increased spatial performance of a cognitive task.[7] Although this paper-and-pencil task tested mental rotation and manipulation of two-and three-dimensional objects, it did not validate the idea that forced right nostril-breathing increased verbal performance on a task modeled after the Miller Analogies and SAT tests. Perhaps the difference in results obtained with normal breathing and with forced uninostril breathing may be related to the fact that one is spontaneous while the other is forced.

The present study aimed to determine whether the nostril which is dominant at rest (i.e., during spontaneous breathing) influences the performance of a task with verbal information that is believed to be a function of the left hemisphere.

## MATERIALS AND METHODS

### Subjects

There were 289 normal healthy volunteers attending a one-week, nonresidential yoga camp. Their age range was from 10 to 79 years (group mean age ± SD, 25.6 ± 14.5 years) with 12 females in the group. Participants who were right hand-dominant were included in the study based on the Edinburgh Handedness Inventory.[8] All participants were informed about the study and their signed informed consent was obtained. Participants were checked for the following conditions: abnormalities of the nasal cavity such as nasal septal deviation or nasal polyps, use of medication which could influence autonomic function (e.g., phenylpropanolamine as a common cold remedy) or an upper respiratory tract infection that could cause nasal blockage. None of the participants had to be excluded based on these exclusion criteria.

### Design

The design included a single assessment within a 30 minute period in the morning; nostril dominance was assessed as described below. After this, participants were given a verbal task (letter cancellation) and nostril dominance was checked once more. Only those participants whose dominant nostril was the same in both assessments, were included in the study.

### Assessment

The letter cancellation task consists of an answer sheet with 24 printed rows and 29 letter in each row. The letter that had to be cancelled within four minutes wherever it appeared in any given row, was indicated in a box at the extreme left (beginning) of each row. Variables belonged to three categories: (1) omitted letters, (2) wrongly cancelled letters, and (3) total errors (i.e., the sum of letters that were left out and wrongly cancelled). After each cancellation task, the nostril dominance was determined by the Zwaardemaker method by using a mirror to measure the right-to-left condensation patterns upon exhalation.[9] This method enables the condensation of the dominant nostril to be larger and remain visible for longer durations.

### Data Analysis

The letters that were left out and wrongly cancelled as well as total errors (i.e., the sum of letters left out and letters wrongly cancelled) were assessed for each participant. The Mann-Whitney u test was used to assess whether there was a significant difference in cancellation task performance between right and left nostril-dominant persons.

In addition to this analysis, the data were also stratified into two groups based on age: (i) age ≤ median age (19 years), and (ii) age > 19 years. Comparisons were made between left and right nostril-dominant participants in both groups, for all three variables (i.e., omitted letters left out, wrongly cancelled letters, and total errors).

Also, irrespective of nostril dominance, all three variables were sorted in ascending order and the median found for each variable. Data less than the median were separated from data greater than the median for all three variables. For each variable, the numbers of participants whose performance was greater or less than the median as well as numbers of left or right nostril-dominant were compared. Separate 2 × 2 Chi-Square tests were done for each variable to compare the numbers of participants who were right nostril-dominant with those who were left nostril-dominant, for values that were greater than and less than the median.

## RESULTS

### Data of the group as a whole

There were no significant differences between the scores for omitted letters (P = 0.867, Mann-Whitney u test), wrongly cancelled letters (P = 0.802) and total errors (P = 0.958) between left and right nostril-dominant participants.

### Data of two groups (Group I with age ≤ median age; Group II with age > median age

**Group I (*n* = 214):** There were no significant differences between the three scores (mentioned above) for participants who were left nostril and right nostril-dominant (P > 0.05, Mann-Whitney u test for all comparisons).

**Group II (*n* = 75):** For this group also, there were no significant differences between the three scores for participants who were left nostril and right nostril-dominant (*P* > 0.05, Mann-Whitney u test for all comparisons).

### Separately stratified data for each of the three variables

The data for the three variables were each separately stratified as those less than and those greater than the median (score). Within a stratum, there were no significant differences between the scores of left and right nostril-dominant participants (P > 0.05, Mann-Whitney u test). 2 × 2 Chi-Square tests also showed no difference based on nostril dominance (P > 0.05).

**Figure 1:**
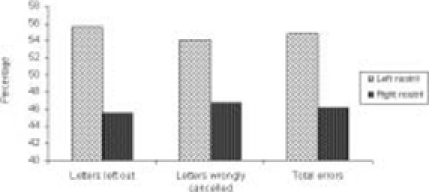
Percentage of subjects who had lower error scores (i.e., values less than the median)

**Figure 2:**
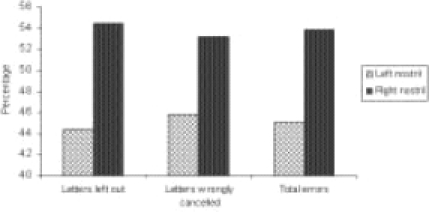
Percentage of subjects who had higher error scores (i.e., values greater than the median)

The group mean values ±S.D. are given in Table 1. The differences between scores of left nostril dominant participants and right nostril dominant participants were not statistically significant, however certain trends have been shown in Figures 1 and 2. This was a trend of a higher percentage of right nostril dominant participants showing more errors compared with the percentage of left nostril dominant persons is shown in Figure 1 (for scores less than the median) and Figure 2 (for scores greater than the median).

**Table 1:** Scores for omitted letters, wrongly cancelled letters, and total errors for right and left nostril-dominant persons. Values are group mean ± SD

Variables	Right nostril	Left nostril
Letters left out	5.87 ± 11.17	7.59 ± 14.89
Letters wrongly cancelled	0.38 ± 1.36	0.31 ± 1.11
Total errors	6.22 ± 11.59	7.90 ± 15.38

## DISCUSSION

There were no significant dominant nostril-based differences in the performance of the letter cancellation task among 289 persons.

Paper-and-pencil tests require rapid visual selectivity while practising a motor response task. These tests assess many functions, not the least of which is the capacity for sustained attention. Visual scanning as well as the activation and inhibition of rapid responses, are also necessary for the successful performance of cancellation tasks. Lowered scores on these tasks can reflect a general slowing of responses and inattentiveness, or more specific defects of response shifting and motor slowing, or of unilateral inattention. With the addition of a motor component, these tasks call upon a set of functions similar to those relevant to other complex tests of attention.[10] The basic format for these tests follows the vigilance test pattern.

Right nostril dominance is believed to be associated with left hemisphere dominance which is connected with verbal task performance. Hence, right nostril breathing may have been expected to be associated with a decrease in errors in a verbal cancellation task. This was not seen in the present study. It has been proposed that it is possible to selectively activate the cerebral hemispheres, thereby enhancing lateralized cognitive abilities. A proposed method of achieving selective activation is by altering nasal congestion/decongestion (nasal cycle), which is believed to effect a contralateral change in hemispheric activation through the autonomic nervous system. Cortical activation and laterality were examined using ratios of the low beta (12-18 Hz) and high alpha (10-12 Hz) bandwidths relative to each other and between hemispheres.[11]

Repeated measures ANOVAs showed non-significant changes in the alpha and beta bandwidths across the 4 experimental conditions. Although changes in hemispheric activation have been postulated for all subjects, the study did not support such changes in subjects untrained in breathing techniques.

Hence this study also did not support the idea of asymmetric hemispheric activation in untrained subjects. However, an earlier study assessed the temporal stability of alpha asymmetry, and examined the relationship between asymmetry and emotion (both state and trait).[12] The findings indicated that the temporal stability of alpha asymmetry was of modest strength, and lower than would be anticipated for an individual difference variable.

The predicted relationship between alpha asymmetry and nostril dominance was found for spontaneous, but not for forced unilateral nostril breathing.

In summary, the present study on 289 participants showed no direct association between the dominant nostril during spontaneous breathing and the performance in a verbal task. However, the study sample had a wide age range and unequal numbers of males and females. Further research should be carried out examining larger samples within a smaller age range and with comparable numbers of males and females.
